# Oxidative Stress: Harms and Benefits for Human Health

**DOI:** 10.1155/2017/8416763

**Published:** 2017-07-27

**Authors:** Gabriele Pizzino, Natasha Irrera, Mariapaola Cucinotta, Giovanni Pallio, Federica Mannino, Vincenzo Arcoraci, Francesco Squadrito, Domenica Altavilla, Alessandra Bitto

**Affiliations:** ^1^Department of Clinical and Experimental Medicine, University of Messina, Messina, Italy; ^2^Department of Biomedical Sciences, Dentistry and Morphological and Functional Images, University of Messina, Messina, Italy

## Abstract

Oxidative stress is a phenomenon caused by an imbalance between production and accumulation of oxygen reactive species (ROS) in cells and tissues and the ability of a biological system to detoxify these reactive products. ROS can play, and in fact they do it, several physiological roles (i.e., cell signaling), and they are normally generated as by-products of oxygen metabolism; despite this, environmental stressors (i.e., UV, ionizing radiations, pollutants, and heavy metals) and xenobiotics (i.e., antiblastic drugs) contribute to greatly increase ROS production, therefore causing the imbalance that leads to cell and tissue damage (oxidative stress). Several antioxidants have been exploited in recent years for their actual or supposed beneficial effect against oxidative stress, such as vitamin E, flavonoids, and polyphenols. While we tend to describe oxidative stress just as harmful for human body, it is true as well that it is exploited as a therapeutic approach to treat clinical conditions such as cancer, with a certain degree of clinical success. In this review, we will describe the most recent findings in the oxidative stress field, highlighting both its bad and good sides for human health.

## 1. Introduction

Superoxide radicals (O_2_^•−^), hydrogen peroxide (H_2_O_2_), hydroxyl radicals (•OH), and singlet oxygen (^1^O_2_) are commonly defined reactive oxygen species (ROS); they are generated as metabolic by-products by biological systems [1, 2]. Processes, like protein phosphorylation, activation of several transcriptional factors, apoptosis, immunity, and differentiation, are all dependent on a proper ROS production and presence inside cells that need to be kept at a low level [3]. When ROS production increases, they start showing harmful effects on important cellular structures like proteins, lipids, and nucleic acids [4]. A large body of evidences shows that oxidative stress can be responsible, with different degrees of importance, in the onset and/or progression of several diseases (i.e., cancer, diabetes, metabolic disorders, atherosclerosis, and cardiovascular diseases) [5].

ROS are mainly produced by mitochondria, during both physiological and pathological conditions, that is, O_2_^•−^ can be formed by cellular respiration, by lipoxygenases (LOX) and cyclooxygenases (COX) during the arachidonic acid metabolism, and by endothelial and inflammatory cells [6]. Despite the fact that these organelles have an intrinsic ROS scavenging capacity [7], it is worth to note that this is not enough to address the cellular need to clear the amount of ROS produced by mitochondria [8].

Cells deploy an antioxidant defensive system based mainly on enzymatic components, such as superoxide dismutase (SOD), catalase (CAT), and glutathione peroxidase (GPx), to protect themselves from ROS-induced cellular damage [9].

## 2. Oxidants and Free Radical Production

ROS production basically relies on enzymatic and nonenzymatic reactions. Enzymatic reactions able to generate ROS are those involved in respiratory chain, prostaglandin synthesis, phagocytosis, and cytochrome P450 system [10–20]. Superoxide radical (O_2_^•−^) is generated by NADPH oxidase, xanthine oxidase, and peroxidases. Once formed, it is involved in several reactions that in turn generate hydrogen peroxide, hydroxyl radical (OH•), peroxynitrite (ONOO^−^), hypochlorous acid (HOCl), and so on. H_2_O_2_ (a nonradical) is produced by multiple oxidase enzymes, that is, amino acid oxidase and xanthine oxidase. Hydroxyl radical (OH•), the most reactive among all the free radical species in vivo, is generated by reaction of O_2_^•−^ with H_2_O_2_, with Fe^2+^ or Cu^+^ as a reaction catalyst (Fenton reaction) [12–19]. Nitric oxide radical (NO•), which plays some important physiological roles, is synthesized from arginine-to-citrulline oxidation by nitric oxide synthase (NOS) [12–19].

Even nonenzymatic reactions can be responsible for free radical production, that is, when oxygen reacts with organic compounds or when cells are exposed to ionizing radiations. Nonenzymatic free radical production can occur as well during mitochondrial respiration [15, 16, 19].

Free radicals are generated from both endogenous and exogenous sources. Immune cell activation, inflammation, ischemia, infection, cancer, excessive exercise, mental stress, and aging are all responsible for endogenous free radical production. Exogenous free radical production can occur as a result from exposure to environmental pollutants, heavy metals (Cd, Hg, Pb, Fe, and As), certain drugs (cyclosporine, tacrolimus, gentamycin, and bleomycin), chemical solvents, cooking (smoked meat, used oil, and fat), cigarette smoke, alcohol, and radiations [15–25]. When these exogenous compounds penetrate the body, they are degraded or metabolized, and free radicals are generated as by-products.

## 3. Physiological Activities of Free Radicals

When maintained at low or moderate concentrations, free radicals play several beneficial roles for the organism. For example, they are needed to synthesize some cellular structures and to be used by the host defense system to fight pathogens. In fact, phagocytes synthesize and store free radicals, in order to be able to release them when invading pathogenic microbes have to be destroyed [16, 21]. The pivotal role of ROS for the immune system is well exemplified by patients with granulomatous disease. These individuals are unable to produce O_2_^•−^ because of a defective NADPH oxidase system, so they are prone to multiple and in most of the cases persistent infections [15, 16]. Free radicals are also involved in a number of cellular signaling pathways [18–20]. They can be produced by nonphagocytic NADPH oxidase isoforms; in this case, free radicals play a key regulatory role in intracellular signaling cascades, in several cell types such as fibroblasts, endothelial cells, vascular smooth muscle cells, cardiac myocytes, and thyroid tissue. Probably, the most well-known free radical acting as a signaling molecule is nitric oxide (NO). It is an important cell-to-cell messenger required for a proper blood flow modulation, involved in thrombosis, and is crucial for the normal neural activity [18]. NO is also involved in nonspecific host defense, required to eliminate intracellular pathogens and tumor cells. Another physiological activity of free radicals is the induction of a mitogenic response [18, 19]. Summarizing, free radicals, when maintained at low or moderate levels, are of crucial importance to human health.

## 4. Detrimental Effects of Free Radicals on Human Health

As stated before, if in excess, free radicals and oxidants give rise to a phenomenon known as oxidative stress; this is a harmful process that can negatively affect several cellular structures, such as membranes, lipids, proteins, lipoproteins, and deoxyribonucleic acid (DNA) [16–21]. Oxidative stress emerges when an imbalance exists between free radical formation and the capability of cells to clear them. For instance, an excess of hydroxyl radical and peroxynitrite can cause lipid peroxidation, thus damaging cell membranes and lipoproteins. This in turn will lead to malondialdehyde (MDA) and conjugated diene compound formation, which are known to be cytotoxic as well as mutagenic. Being a radical chain reaction, lipid peroxidation spreads very quickly affecting a large amount of lipidic molecules [25]. Proteins may as well being damaged by oxidative stress, undergoing to conformational modifications that could determine a loss, or an impairment, of their enzymatic activity [20, 25].

Even DNA is prone to oxidative stress-related lesions, the most representative of which is the 8-oxo-2′-deoxyguanosine (8-OHdG) formation; this is a particularly pernicious DNA lesion, which can be responsible for both mutagenesis, as pointed out by Nishida et al. [26]. It can also cause a loss in the epigenetic information, probably due to an impairment in CpG island methylation asset in gene promoters [27]. It is worth to note that Valavanidis and colleagues [28] have already proposed 8-OHdG levels in a tissue as biomarker of oxidative stress. Of course cells can put in place several mechanisms, such as the base excision repair (BER) or antioxidants, as defense response against DNA lesions [17–20].

If not strictly controlled, oxidative stress can be responsible for the induction of several diseases, both chronic and degenerative, as well as speeding up body aging process and cause acute pathologies (i.e., trauma and stroke).

### 4.1. Cancer and Oxidative Stress

Cancer onset in humans is a complex process, which requires both cellular and molecular alterations mediated by endogenous and/or exogenous triggers. It is already well known that oxidative DNA damage is one of those stimuli responsible for cancer development [14, 15, 22]. Cancer can be driven and/or promoted by chromosomal abnormalities and oncogene activation determined by oxidative stress. Hydrolyzed DNA bases are common by-products of DNA oxidation and are considered one of the most relevant events in chemical carcinogenesis [14, 22]. The formation of such kind of adducts impairs normal cell growth by altering the physiological transcriptomic profile and causing gene mutations. Oxidative stress can also cause a variegated amount of modifications against DNA structure, for example, base and sugar lesions, DNA-protein cross-links, strand breaks, and base-free sites. For instance, tobacco smoking, environmental pollutants, and chronic inflammation are sources of oxidative DNA damage that could contribute to tumor onset [14, 17, 29]. Oxidative stress resulting from lifestyle reasons can also play an important role in cancer development, as suggested by the strong correlation between dietary fat consumption (a factor that exposes the organism at greater risk of lipid peroxidation) and death rates from different types of cancer [16, 21].

### 4.2. Cardiovascular Disease and Oxidative Stress

Cardiovascular diseases (CVDs) are clinical entities with a multifactorial etiology, generally associated with a very large amount of risk factors, the most broadly recognized of which are hypercholesterolaemia, hypertension, smoking habit, diabetes, unbalanced diet, stress, and sedentary life [11, 30, 31]. During the last years, research data pointed out that oxidative stress should be considered either a primary or a secondary cause for many CVDs [18]. Oxidative stress acts mainly as a trigger of atherosclerosis. It is well known that atheromatous plaque formation results from an early endothelial inflammation, which in turn leads to ROS generation by macrophages recruited in situ. Circulating LDL are then oxidized by reactive oxygen species, thus leading to foam cell formation and lipid accumulation. The result of these events is the formation of an atherosclerotic plaque. Both in vivo and ex vivo studies provided evidences supporting the role of oxidative stress in atherosclerosis, ischemia, hypertension, cardiomyopathy, cardiac hypertrophy, and congestive heart failure [11, 16, 30, 31].

### 4.3. Neurological Disease and Oxidative Stress

Oxidative stress has been linked to several neurological diseases (i.e., Parkinson's disease, Alzheimer's disease (AD), amyotrophic lateral sclerosis (ALS), multiple sclerosis, depression, and memory loss) [32–35]. In AD, several experimental and clinical researches showed that oxidative damage plays a pivotal role in neuron loss and progression to dementia [34]. *β*-amyloid, a toxic peptide often found present in AD patients' brain, is produced by free radical action and it is known to be at least in part responsible for neurodegeneration observed during AD onset and progression [35].

### 4.4. Respiratory Disease and Oxidative Stress

Several researches pointed out that lung diseases such as asthma and chronic obstructive pulmonary disease (COPD), determined by systemic and local chronic inflammation, are linked to oxidative stress [36–39]. Oxidants are known to enhance inflammation via the activation of different kinases involving pathways and transcription factors like NF-kappa B and AP-1 [38, 39].

### 4.5. Rheumatoid Arthritis and Oxidative Stress

Rheumatoid arthritis is a chronic inflammatory disorder affecting the joints and surrounding tissues, characterized by macrophages and activated T cell infiltration [15, 40, 41]. Free radicals at the site of inflammation play a relevant role in both initiation and progression of this syndrome, as demonstrated by the increased isoprostane and prostaglandin levels in synovial fluid of affected patients [41].

### 4.6. Kidney Diseases and Oxidative Stress

Oxidative stress is involved in a plethora of diseases affecting renal apparatus such as glomerulo- and tubule-interstitial nephritis, renal failure, proteinuria, and uremia [16, 42]. The kidneys are negatively affected by oxidative stress mainly because of the fact that ROS production induces the recruitment of inflammatory cells and proinflammatory cytokine production, leading to an initial inflammatory stage. In this early phase, a predominant role is played by TNF-alpha and IL-1b, as proinflammatory mediators, as well as by NF-*κ*B as transcriptional factor required to sustain the inflammatory process. The latter stage is characterized by an increase in TGF-beta production, which orchestrates the extracellular matrix synthesis. So, when the oxidative stress stimuli act chronically on kidney tissues, the results will be an initial stage of inflammation and later the formation of abundant fibrotic tissue that impairs organ function potentially leading to renal failure. Certain drugs, such as cyclosporine, tacrolimus, gentamycin, and bleomycin, are known to be nephrotoxics mainly because of the fact that they increase free radical levels and oxidative stress via lipid peroxidation [42–45]. Heavy (Cd, Hg, Pb, and As) and transition metals (Fe, Cu, Co, and Cr), acting as powerful oxidative stress inducers, are responsible for various forms of nephropathy, as well as for some types of cancers [22, 23].

### 4.7. Sexual Maturation and Oxidative Stress

Several authors pointed out that oxidative stress could be responsible for a delayed sexual maturation and puberty onset [46, 47]. This seems to be true when children in prepubertal age are exposed to Cd, a well known responsible for an increase in free radicals and oxidative stress, as well as when pregnant women are exposed to the same metallic element.

Summarizing, we can affirm that oxidative stress and free radicals are confirmed to be responsible for several pathological conditions affecting different tissues and systems, thus being one of the most important and pervasive harms to human health.

## 5. Exogenous Antioxidants and Human Health

Human body put in place several strategies to counteract the effects of free radicals and oxidative stress, based on enzymatic (e.g., SOD, CAT, and GPx) and nonenzymatic (e.g., lipoic acid, glutathione, ʟ-arginine, and coenzyme Q10) antioxidant molecules, all of them being endogenous antioxidants. Beside these, there are several exogenous antioxidant molecules of animal or vegetal origin, mainly introduced by diet or by nutritional supplementation.

Here, we will discuss the most relevant nutritional antioxidants and their protective effects for human health.

### 5.1. Vitamin E

The term vitamin E encompasses a constellation of lipophilic molecules (*α*-, *β*-, *γ*-, and *δ*-tocopherol and *α*-, *β*-, *γ*-, and *δ*-tocotrienol) synthesized by vegetal organisms [48] and contained in edible oils and seeds, as well as in food artificially enriched in *α*-tocopherol [49, 50].

The most active form of vitamin E, RRR-*α*-tocopherol, showed in vitro an antiproliferative activity against vascular smooth muscle cells via PKC modulation [51], even when under stimulation from low-density lipoproteins (LDL) [52]. These results were confirmed in vivo, both in mouse and rabbit models of atherosclerosis [53–55].

Macrophage transition to foam cells is one of the earlier and important steps in atherosclerotic lesion formation; CD36 receptor is one of the key players involved, being a scavenger receptor responsible for oxidized-LDL (oxLDL) uptake from bloodstream [56, 57].

Several studies described that vitamin E is able to prevent CD36 mRNA expression induced by cholesterol, thus playing a beneficial role in preventing foam cell formation. This was true in vivo, as well as in vitro on human macrophages and vascular smooth muscle cells [58, 59]; vitamin E supplementation was also useful to upregulate PPAR*γ*, LXR*α*, and ABCA1, in ApoE knockout mice, ameliorating early (but not advanced) atherosclerotic lesions [60].

Vitamin E modulates the oxidative stress-induced NF-*κ*B pathway and oxLDL-induced foam cell formation, decreases c-Jun phosphorylation (thus inhibiting inflammation and monocyte invasion), and matrix metalloprotease (MMP) expression [61–65].

A degree of CD36 mRNA reduction was also observed in animals undergoing to vitamin E supplementation under a regimen of high-fat diet [66–68].

RRR-*γ*-tocopherol (the most abundant after RRR-*α*-tocopherol) showed a potent proinflammatory function during allergic inflammation [69–77].

Each form of vitamin E seems to have different regulatory effects when it comes to recruit leukocytes to allergic inflammation site, which is however strictly dependent on vascular cell adhesion molecule-1 (VCAM-1) [78]. VCAM-1 is responsible also for the activation of several signals in endothelial cells which are causative of ROS generation, such as NOX2 complex activation that generates ROS which lead to PKC*α* activation [79]. This rapid and transient PKC*α* activation is consistent with a leukocyte migration across endothelia required in a timeframe of minutes, consistent with the rapid migration of leukocytes across endothelial cells in minutes at sites of migration.

Endothelial cells pretreated with *α*-tocopherol are less prone to let the lymphocytes and eosinophils migrate, while the opposite is true when pretreated with *γ*-tocopherol, this is due to the fact that the first strategy decreases VCAM-1 expression, while the latter increases it [80]. This phenomenon was observed even in vivo, in a mouse model of allergic lung inflammation [80, 81].

Interestingly, a research found a correlation between the prevalence of asthma and the average plasma tocopherol in several countries, based on nutritional consumption of foods and oils rich in tocopherol. Briefly, countries with an average plasma *γ*-tocopherol concentration of 2–7 *μ*mol/L had the highest asthma prevalence compared to those with a concentration of 1-2 *μ*mol/L, independently from *α*-tocopherol plasma levels [74].

Olive and sunflower oils, which have little or not at all *γ*-tocopherol [74], seem to have to be preferred to soybean oil, because the latter one seems responsible for an increase in plasma *γ*-tocopherol [82].

A large prospective study, covering 4500 individuals and spanning 20 years, demonstrated an association between *α*- or *γ*-tocopherol serum concentrations and lung function [72]. The results highlighted as in those individuals with higher *γ*-tocopherol serum levels (>10 *μ*mol/L) demonstrated significantly lower FEV1/FVC (10–17%); it is relevant to point out that similar degrees of lung function impairments were observed in individuals exposed to other respiratory stressors (e.g., particulate matter) [83–87]. These observations suggest that *γ*-tocopherol could negatively affect pulmonary function.

It has been also observed, from in vivo experiments, that *α*- and *γ*-tocopherol supplementation of allergic and nonallergic pregnant mice can alter the allergic responsiveness development in offspring of mice.

It is known that (i) proper dendritic cell development and responsiveness are crucial for an optimal allergen sensitization, (ii) it relies on PKC isoforms activity, and (iii) all of the PKCs include a C1A regulatory domain, which is targeted by both *α*- and *γ*-tocopherol [88–107].

In mice prone to allergic disease, supplementing allergic mothers (at the time of mating) with *α*-tocopherol was enough to inhibit the pup allergic responses [67], while *γ*-tocopherol supplementation amplified pup responses to allergens [70].

These differences in allergic response development exerted by *α*- or *γ*-tocopherol supplementation are dependent from their modulation of eosinophils and CD11c^+^ CD11b^+^ dendritic cell numbers in the lungs; *α*-tocopherol reduced both the cellular species, without affecting the number of CD11c^+^ CD11b^−^ regulatory dendritic cells, while *γ*-tocopherol increased both eosinophils and CD11c^+^ CD11b^+^ dendritic cells [69, 70].

Summarizing, *α*-and *γ*-tocopherol forms of vitamin E exert a differential set of biological effects, which cannot be always regarded as positive to human health; this is something that needs to be taken in account when considering to enrich the content of vitamin E into a diet with antioxidant purposes.

### 5.2. Flavonoids

Flavonoids are a class of polyphenolic compounds with a benzo-*γ*-pyrone structure largely represented in plants, responsible for several pharmacological activities [108, 109]. These substances have been investigated because of their potential health benefits as antioxidants, action mediated by their functional hydroxyl groups, which are able to scavenge free radicals and/or chelate metal ions [109–116].

Their antioxidant activity relies on the conformational disposition of functional groups; configuration, substitution, and total number of hydroxyl groups are important factors in determining mechanisms of antioxidant activity like ROS/RNS scavenging and metal chelation [111, 117].

Flavonoid determines (i) ROS synthesis suppression, inhibition of enzymes, or chelation of trace elements responsible for free radical generation; (ii) scavenging ROS; and (iii) improvement of antioxidant defenses [118, 119].

Genistein is a soy isoflavone that is probably the most interesting and well-studied flavonoid compound, due to its broad pharmacological activities.

Genistein has been extensively employed as antioxidant in a plethora of studies, showing the potential to scavenge ROS and RNS with a high degree of efficacy. This flavonoid compound is able to improve the antioxidant defenses of a cell, thus prevents apoptotic process through the modulation of several genes and proteins [120]. In nonhuman primates and rabbits [121, 122], dietary-supplemented genistein delayed atherogenesis. An additional study observed an increase in antioxidant protection of LDL and an atheroprotective effect [123]. In general, soy isoflavones confer protection against lipoprotein oxidation [124–126], as well as against oxidative DNA damage in postmenopausal women [127], but the point is still debated [128–130]. There are other mechanism that genistein can be used to suppress oxidative stress and related inflammation in the vascular intima layer. Genistein inhibits NF-*κ*B activation (inducible by oxidative stress) and regulates the expression of genes relevant to immune and inflammatory processes [131]. Genistein increases the expression of antioxidant enzymes in human prostate cancer cells conferring protection against oxidative DNA damage [132, 133].

Briefly, flavonoids are a class of natural compounds extensively present in foods of vegetal origin (fruits, oils, seeds, etc.) showing a good potential in terms of usefulness for human health, as antioxidant molecules but also because of some ancillary yet pharmacologically interesting properties. Nonetheless, they need to be managed carefully, and their supplementation into the diet (as diet enrichment or as nutraceuticals) have to take in account also some potential drawback concerning human health and wellness.

## 6. Prooxidant Agents in Therapy

Prooxidant agents, beside their well-known detrimental effects on human health, have been investigated and, in some cases, actually used, as therapeutic agents mainly against cancer diseases.

Here, we will briefly discuss two emerging prooxidant compounds showing interesting pharmacological activities, such as ascorbic acid (AA) and polyphenols, and the most well-known and employed prooxidant in therapy, ionizing radiation.

### 6.1. Ascorbic Acid

Ascorbic acid (vitamin C) is a water-soluble compound classified under the group of natural antioxidants. Ascorbate reacts with ROS, quenching them and promoting the conversion into semihydroascorbate radical, which is a poorly reactive chemical species, thus efficiently reducing the risk of cancer by suppressing free radicals and oxidative stress [134].

Apart from this, ascorbate also reduces metal ions like Fe^3+^ and Cu^3+^, thus promoting a reaction that gives rise to highly reactive free radical (by the so-called Fenton reaction) [135, 136]; these radicals have been reported to be able to induce cytotoxicity by causing DNA backbone breaks and base modifications [134].

This effect seems to be more relevant on cancer cells, in fact while normal cells take advantage from redundant mechanisms for H_2_O_2_ clearing and/or repair of H_2_O_2_-induced damage, to counteract the effects of pro-oxidant concentrations of AA; cancer cells lacking of these compensatory mechanisms (e.g., catalase deficiency, mutated DNA repair, and tumor suppressor genes) are more susceptible to pharmacologic ascorbate concentrations [132]. The authors reported that 10 mM AA induces apoptosis in leukemia cell lines; the authors proposed that AA-induced O_2_^•−^/H_2_O_2_ production led to NF-*κ*B-p53-caspase 3 signaling axis, by which this proapoptotic effect is exerted [137–139]. Another study pointed out that AA was able to inhibit Raji cell proliferation, apparently by downregulating the set of genes needed for S-phase progression in actively proliferating cells [140]. In an in vivo study, guinea pigs supplemented with AA at various doses showed a complete regression of fibrosarcoma and liposarcoma tumors [141]. In general, there have been several studies assessing the antiblastic activities of AA, mostly in vitro on different cell lines [142–156].

Despite these somehow surprising but still very interesting results, there is the urge of conducting more researches, both in vitro and in vivo, to definitely assess the mode of action and efficacy of AA as prooxidative anticancer agent.

### 6.2. Polyphenols

Under conditions like high concentrations, high pH, and the presence of redox-active metals, phenolic compounds can acquire a prooxidant behavior [157, 158], mainly based on the generation of an aroxyl radical or a labile complex with a metal cation exerting redox activity. Aroxyl radical can lead the formation of O_2_^•-^ or of ternary compound between DNA, copper, and flavonoids [159]. Polyphenols, like caffeic acid, ferulic acid, and apigenin, can exert a prooxidant effect through the increased intracellular production of ROS by NOX [160, 161].

Polyphenols can as well induce oxidative stress via transition metals, promoting the generation of hydroxyl radicals through Fenton and Fenton-like reactions; it is important to note that transition metal ions are more represented into cancer than into normal cells [162].

Prooxidant polyphenols seem to exert their cytotoxic activity by inducing apoptosis and cell cycle arrest via several pathways. Anthocyanins, pigments present in red wine and berry (*Aronia melanocarpa*, Rosaceae, *Vaccinium myrtillus*, and Ericaceae) fruits, cause apoptosis in cancer cells by increasing intracellular ROS formation [162–164].

Esculetin, a coumarin derivative present in plants such as chicory (*Cichorium intybus* and Asteraceae), showed both in vivo and in vitro antiproliferative activity against hepatocellular carcinoma. Esculetin delay Hepa 1–6 cell growth inoculated subcutaneously in C57BL/6J mice in a time- and dose-dependent manner [165]. Human hepatocellular carcinoma SMMC-7721 cells incubated with esculetin undergo to mitochondrial membrane potential collapse, with Bcl-2, caspase-9-, and caspase-3-mediated apoptosis [165]. In addition, esculetin also exerted a cytotoxic effect on HeLa cells inducing redox-dependent apoptosis, even in this case by causing the disruption of mitochondrial membrane potential, cytochrome C release, and caspase activation [166].

Curcumin, a compound extracted from *Curcuma longa*, induced ROS-mediated apoptosis in human gastric BGC-823 cells by activating the apoptosis signal-regulating kinase 1 (ASK1) signaling cascade (ASK1/MKK4/JNK) [167].

During the last years, a very large amount of in vitro studies investigated the prooxidative effects of polyphenols against cancer cell proliferation and survival, all of them presenting interesting results that nonetheless need to be confirmed by more in-depth researches [168–190].

Although polyphenols showed the pharmacological potential to inhibit tumorigenesis and arrest cancer cell proliferation in animal models, the role of ROS generation is still poorly understood, mainly because a large majority of the in vivo studies are limited to cancer growth arrest and apoptosis evaluation, and rarely or not at all they go deeper in the mechanistic explanation of a potential prooxidant action in vivo [191, 192].

### 6.3. Radiation Therapy

The ability of ionizing radiation to counteract proliferation of cancer cells is well explained [193–195] and widely used in clinical practice. In the last decades, there has been an extensive effort to understand the physical and molecular cellular response that follow the exposure to ionizing radiation. It is well recognized that damage to DNA operated by generation of radicals that indirectly cause DNA double-strand beaks (DSBs) is the most severe kind of damage induced by this prooxidant physical agent [196, 197]. These lesions are promptly repaired, as the results of the rapid activation of DSB damage repair mechanism, most importantly nonhomologous end joining or homologous recombination and the execution of a complex and finely tuned sequelae of those cellular signaling pathways belonging to the DNA damage response (DDR) [194, 198]. These responses span from posttranslational modifications and/or differential gene expression of proteins to start cell cycle reprogramming (e.g., radiation-induced arrest) or to execute cell death by mitotic catastrophe, apoptosis, autophagy, or induction of senescence [194, 195, 198].

Radiotherapy plays a key role in cancer treatment, so that almost 40% of cancer patients have been treated with this approach at least once [199]. In the last 2 decades, several technological advancements, like intensity-modulated radiotherapy (IMRT), image-guided radiotherapy (IGRT), and stereotactic radiotherapy (SRT), were put in place to address the need to reach that level of precision required to take advantage from radiation prooxidant activity avoiding, as much as possible, the side effects in terms of oxidative stress-induced cellular damage on healthy cells and tissues.

## 7. Conclusions

Oxidative stress and free radicals are generally known to be detrimental to human health. A large amount of studies demonstrates that in fact free radicals contribute to initiation and progression of several pathologies, ranging from CVD to cancer.

Antioxidants, as class of compounds able to counteract oxidative stress and mitigate its effects on individuals' health, gained enormous attention from the biomedical research community, because these compounds not only showed a good degree of efficacy in terms of disease prevention and/or treatment but also because of the general perception that they are free from important side effects. If it is true that antioxidants can be very useful in preventing, managing, or treating human pathologies, it is true as well that they are not immune to generating adverse effects. On the other hand, some prooxidant compounds or agents can be as well useful to human health, particularly regarding cancer treatment.

We can reach to the conclusion that oxidative stress, as phenomenon, although being one of the major harms to individuals' wellness and health, it can also be exploited as a treatment tool when and if we will be able to operate a fine tuning of this process inside human organism.
